# Commencements

**Published:** 1895-07

**Authors:** 


### COMMENCEMENTS.

### Baltimore College of Dental Surgery.

The fifty-fifth annual commencement exercises of the Baltimore College of Dental Surgery were held at Ford's Grand Opera House, Baltimore, Md., on Thursday afternoon, March 21, 1895, at 2 o'clock.

The annual oration was delivered by Rev. William Rollins Webb, and the valedictory by B. M. Smith, D.D.S.

The number of matriculates for the session was one hundred and sixty-six.

The degree of D.D.S. was conferred on the following graduates by Professor M. W. Foster, Dean:

| NAME. | RESIDENCE. |
|---|---|
| M. H. Adams | Louisiana |
| J. G. Armacost | Maryland |
| H. G. Baxter | New York |
| H. J. Boyd | New York |
| C. E. Brown | Virginia |
| S. P. Cronan | Connecticut |
| W. G. Dalzell | Louisiana |
| G. R. Dashiell | Texas |
| A. E. DeViney | Texas |
| H. R. Eavey | Maryland |
| K. W. Egerton | Maryland |
| F. W. Epes | Virginia |
| C. H. Frink | Florida |
| J. G. Geiger | South Carolina |
| Fred. Hammond | Maryland |
| D. S. Henry | South Carolina |
| M. J. Hellwig | New York |
| H. Hoeper | Germany |
| H. W. Hoffer | Texas |
| E. G. Laflin | Connecticut |
| T. H. Lowe | Massachusetts |
| J. W. Lyle | Mississippi |
| A. M. Marcy | Ohio |
| C. Meikle | New York |
| O. Morgan | Pennsylvania |
| J. B. Rousseau | Alabama |
| L. D. Reavis | Oregon |
| P. C. Richardson | Texas |
| I. C. Rink | Pennsylvania |
| W. R. Roland | Pennsylvania |
| W. H. Simpson | Massachusetts |
| G. F. Showers | West Virginia |
| B. M. Smith | Pennsylvania |
| E. M. Summerville | Pennsylvania |
| E. H. Sting | Ohio |
| W. M. Sturgis | Virginia |
| C. H. Theberath | New Jersey |
| A. C. Thweatt | Virginia |
| F. Waesche | Maryland |
| J. L. Walker | Virginia |
| R. H. Weiskotten | New York |
| E. Weymouth | Maine |
| G. W. Williams | West Virginia |
| J. H. Wheeler | North Carolina |
| H. White | Maryland |

### Ohio College of Dental Surgery.

The forty-ninth annual commencement exercises of the Ohio College of Dental Surgery (Department of Dentistry of the University of Cincinnati) were held in the Auditorium of the Odd Fellows' Temple, Cincinnati, Ohio, on Tuesday, April 2, 1895, at 8 o'clock P. M.

The annual address was delivered by Dr. W. O. Thompson, president of Miami University, and the class oration by Charles E. Fitzpatrick, D.D.S.

The number of matriculates for the session was one hundred and eighty-nine.

The degree of D.D.S. was conferred on the following graduates by James Leslie, D.D.S., of the board of trustees:

| NAME. | RESIDENCE. |
|---|---|
| F. P. Anshutz | Indiana |
| W. G. Baker | New York |
| C. H. Barlow | Ohio |
| A. P. Bell | Ohio |
| E. M. Revard | Ohio |
| H. J. Bond | Pennsylvania |
| J. W. Bond | Indiana |
| W. C. Bowyer | Ohio |
| A. B. Boyd | Kentucky |
| H. H. Braxtan | Indiana |
| J. A. Calhoun | Ohio |
| A. P. Chambers | Ohio |
| A. H. Clancey. | Ohio |
| F. A. Couch | West Virginia |
| E. M. Davis | Pennsylvania |
| C. O. Edwards | Indiana |
| H. D. Finney | Pennsylvania |
| C. E. Fitzpatrick | Ohio |
| J. E. Froendhoff | Ohio |
| H. Gillham | Kentucky |
| W. Gillham | Kentucky |
| J. B. Griffiths | Ohio |
| W. P. Huston | Ohio |
| W. H. Johnson | Ohio |
| D. P. Jones | Ohio |
| C. C. Leming | Indiana |
| E. Matthews | Kentucky |
| F. E. McGilliard | Ohio |
| C. P. McLaughlin | Indiana |
| A. J. Myers | Indiana |
| J. N. Myers | Ohio |
| C. M. Meade | Indiana |
| O. Miesse | Ohio |
| T. S. Noble, Jr | Ohio |
| W. H. Rogers | Ohio |
| M. J. Ruddy | Kansas |
| C. A. Porter | Indiana |
| E. Shaw | Ohio |
| R. A. Sprake | Kentucky |
| J. V Stahl | Ohio |
| L. Stern | Ohio |
| H. H. Stevenson | Minnesota |
| T. Storey | Canada |
| F. J. Tarrant | New York |
| F. S. Taylor | Ohio |
| W. J. Thomas | Pennsylvania |
| G. D. Thorne | New York |
| C. D. Van Houten | Ohio |
| A. S. Woodrow | Ohio |
| R. L. Walsh | California |

### Pennsylvania College of Dental Surgery.

The thirty-ninth annual commencement exercises of the Pennsylvania College of Dental Surgery were held at the American Academy of Music, Philadelphia, Penn., on Wednesday evening, March 6, 1895.

The address to the graduates was delivered by J. Ewing Mears, M.D., professor of anatomy and surgery.

The number of matriculates for the session was three hundred and nine.

The degree of D.D.S. was conferred on the following graduates by I. Minis Hays, M.D., president of the board of corporators:

| NAME. | RESIDENCE. | NAME. | RESIDENCE. |
| --- | --- | --- | --- |
| Harry Archy | Pennsylvania | John Krell | Pennsylvania |
| J. S. Ashbrook | Pennsylvania | E. H. Kuhns | Pennsylvania |
| H. P. Bachman | Pennsylvania | W. G. Lowrey | Pennsylvania |
| John C. Bansley | Canada | A. B. Longshore | Pennsylvania |
| Chas. R. Berlew | New Jersey | Chauncy Lunger | Pennsylvania |
| F. Sloan Betts | New York | M. L. Mandelstam | Pennsylvania |
| Teofilo Borrero | U. S. of Colombia | Geo. S. Mason | Pennsylvania |
| E. Lee Boyles | Pennsylvania | L. W. McCullough | Michigan |
| H. M. Brown | Ohio | J. J. F. McLaughlin | Massachusetts |
| Thurl E. Bullard | Maryland | Howard Mellor | Pennsylvania |
| W. P. Clark | Connecticut | H. C. Miller | Pennsylvania |
| E. Clark, L.D.S | England | B. W. Moore | New Jersey |
| Jas. Cleary | Massachusetts | J. W. Mountain | Connecticut |
| A. J. Cottee | Australia | W. F. O'Mally | Massachusetts |
| R. S. Connor | Ohio | Beat'ce F. Osborn | New York |
| Chas. W. Cooke | New Jersey | Seth G. Osborn | New York |
| N. J. Coyne | New York | R. C. Phillips | England |
| J. I. Creasy | Pennsylvania | F. W. Proseus | Rhode Island |
| A. B. Dewees | New Jersey | W. R. Roe | Canada |
| G. W. C. Entelrine | Pennsylvania | O. P. Roller | California |
| J. E. Evans | Ohio | Anne L. Russell | Pennsylvania |
| Carl Fisher | Germany | Severo Salced S | Chile |
| V. P. Ford | Pennsylvania | Carl Scheidt | Germany |
| H. E. Friesell | Pennsylvania | J. W. Shuman | Pennsylvania |
| Alfred Garcia | Cuba | A. G. Slonaker | New York |
| Chas. M. Gowen | Georgia | O. G. Smith | New Jersey |
| S. H. Griffin | Massachusetts | J. F. Smull | Pennsylvania |
| Michael Grady | Connecticut | W. C. Spencer | Pennsylvania |
| Martin Haase | Germany | L. E. Sticker | Pennsylvania |
| J. M. Haymaker | Pennsylvania | H. J. Stewart | Pennsylvania |
| F. A. Haymaker | Pennsylvania | F. A. Stewart | Pennsylvania |
| Elwood Hay | New Jersey | H. J. Steil | New York |
| Geo. W. Hartzel | Pennsylvania | G. H. Thompson | Canada |
| H. G. Holch | New York | R. W. Turner | Pennsylvania |
| W. T. Herbst | Pennsylvania | W. H. Van Meter | New Jersey |
| H. E. Hosley | Pennsylvania | P. S. Voegtlen | New Jersey |
| J. B. Hoffman | Pennsylvania | Edwin G. Warne | England |
| R. W. Hue | England | Geo. L. Wernet | Ohio |
| R. B. Hubbard | New York | H. K. Wilkins | Pennsylvania |
| J. H. Ivins | New Jersey | R. A. Willmott | Canada |
| R. W. Jewett | New Jersey | M. L. Zimmerman | Russia |
| J. C. Kamerley | Pennsylvania | | |

Total 83.

### Philadelphia Dental College.

The thirty-second annual commencement exercises of the Philadelphia Dental College were held at the American Academy of Music, Philadelphia, Pa., on Thursday evening, March 7, 1895.

The address to the graduates was delivered by Professor Thomas C. Stellwagen, and the valedictory address by George Fulton Taylor, D.D.S.

The number of matriculates for the session was three hundred and sixty-seven:

The degree of D.D S. was conferred on the following graduates by Ex-Governor James A. Beaver, president of the board of trustees:

| NAME. | RESIDENCE. |
|---|---|
| Erik G. Akerlund | Sweden |
| Geo. C. Anderson | New York |
| C. U. Anthony | Pennsylvania |
| Fred. Eugene Ball | Maine |
| Arthur Laird Ball | Canada |
| John J. Barrett | Pennsylvania |
| George B. Beach | New York |
| Albert O. Berg | Sweden |
| M. W. Bieberbach | Germany |
| Harvey R. Black | Maryland |
| Charles H. Bogart | New York |
| Frederick S. Brooks | California |
| James B. Caine | New York |
| Richard H. Calely | Pennsylvania |
| C. D. Candler | Maryland |
| H. A. Carothers | Pennsylvania |
| Theo. D. Casto | West Virginia |
| A. D. Coleman | Massachusetts |
| James F. Coll | Pennsylvania |
| M. J. Collins | Maine |
| M. B. Crisman | Pennsylvania |
| Eliz. Cummings | California |
| Mrs. M. E. Cummings | California |
| Willsam S. Dalby | Britsh Columbia |
| A. G. Danforth | Maine |
| Victor E. Darling | Massachusetts |
| Joseph I. DeRoy | Pennsylvania |
| Wardell F. Engle | New York |
| Geo. C. Ferguson | Canada |
| Cassius L. Finney | Pennsylvania |
| R. J. Fitz Simons | Canada |
| David L. Floore | Kentucky |
| Rose Flynn | Pennsylvania |
| John H. Foquet | Pennsylvania |
| John W. Forssell | Utah |
| Harry A. Foreman | Pennsylvania |
| Harry K. Freeman | Pennsylvania |
| Chas. C. Galloway | Iowa |
| H. D. Gihon, Jr | New Jersey |
| Wm. L. Githens | New Jersey |
| James C. Gleason | Rhode Island |
| I. Milton Hadcock | Canada |
| G. S. Hagerthy | Maine |
| Edgar H. Hand | Pennsylvania |
| E. F. Hilferty | Pennsylvania |
| William L. Hill | New York |
| Alvin W. Irving | Texas |
| C. P. Jones, Ph.G | New York |
| A. B. Kelly, B.S | Mississippi |
| John P. Kelly | Massachusetts |
| Edward A. Kent | Michigan |
| H. A. Krumrine | Pennsylvania |
| Robert M. Leslie | Montana |
| Isidore Lett | Pennsylvania |
| F. Lawrason | Canada |
| C. H. MacDonald | Massachusetts |
| George A. Magee | Pennsylvania |
| John W. Martin | New York |
| Josiah Mason | Canada |
| W. J. McElligott | Connecticut |
| T. F. McElligott | Pennsylvania |
| E. McKenney | Canada |
| I. W. E. McLellan | Canada |
| W. W. McVea | Louisiana |
| J. W. Meadows | Canada |
| Glen W. Means | Pennsylvania |
| W. C. Mechem | Kansas |
| W. H. Merrigan | Massachusetts |
| Mrs. M. M. Moyer | Pennsylvania |
| C. R. Murphy | Canada |
| T. J. O'Connor | Massachusetts |
| W. H. Owens | New Jersey |
| Geo. E. Ozias | Pennsylvania |
| Joseph B. Page | Kansas |
| Thomas Parker | Pennsylvania |
| Arthur Pearson | Connecticut |
| W. J. Peebles | Pennsylvania |
| C. F. Pepper | California |
| Louis E. Plant | Connecticut |
| A. D. Preston | Massachusetts |
| A. L. Puckey | Pennsylvania |
| John F. Regan | Massachusetts |
| E. A. Rickards | Maryland |
| Chas. A. Riedel | Germany |
| J. F. Robertson | Washington |
| Peter Rogmans | Netherlands |
| F. B. Rose | New York |
| John F. Ross | Canada |
| Robert E. Rose | Iowa |
| Henry S. Russell | New Jersey |
| C. E. Sawyer | Maine |
| C. H. Scruton | New Hampshire |
| W. F. Shaw | Massachusetts |
| Stella Sims | Illinois |
| D. G. Snyder, B.E | Pennsylvania |
| Rasmus Steensen | Denmark |
| C. E. Stolte | Oregon |
| W. H. Strangways | Canada |
| T. R. Stuart | Canada |
| C. A. Summers | Pennsylvania |
| George F. Taylor | Massachusetts |
| John A. Taylor | Pennsylvania |
| F. J. Tewksbury | Vermont |
| A. C. Thompson | Connecticut |
| W. E. Travers | New York |
| Harry V. Walls | Connecticut |
| C. W. B. Wheeler | New York |
| C. A. Wilbur | Rhode Island |
| E. E. Williams | Pennsylvania |
| James A. Witter | Pennsylvania |
| Walter Wood | Georgia |
| Peter H. Wood | Newfoundland |
| James B. Woods | Pennsylvania |
| J. E. Woodworth | Wisconsin |
| James C. Wright | New Jersey |

Total 115.

### New York College of Dentistry.

The twenty-ninth annual commencement exercises of the New York College of Dentistry were held in Chickering Hall, New York City, on Monday evening, April 15, 1895.

The address to the graduates was delivered by Rev. Geo. R. Van de Water, D.D., and the valedictory by J. Levin Chaim, M.D.S., D.D.S.

The number of matriculates for the session was three hundred and sixty-one.

The degree of D D.S. was conferred on the following graduates by Professor Frank Abbott, M.D., dean of the faculty:

Robert R. Abrams.

Irving F. Adams.

William H. Achtel.

Joseph D. Alvord.

Ralph E. Aitken.

Otto H. Albanesius.

Eugene F. Beers.

Albert R. Benedict.

Arthur I. Bernstein.

Albert R. Brown.

Ernest T. Brown.

Frederick C. Brush.

Joseph L. Chaim, M.D.S.

Myron L. Colburn.

John T. Conlon.

Ernest A. Wm. Dahlman, A.B.

Clinton A. Downs.

William B. Dunning.

Robert McK. Farris.

John T. Gilchrest.

Horace P. Gould.

Solomon R. Halley.

Harry E. Hawes.

Louis A. Held.

Michael P, Higgins.

Charles M. Hoblitzell.

James D. Hyland.

George A. James.

Albert M. Johnston.

Herman J. Kaufer.

John W. Kingsland.

Charles F. Kirkup.

Albert E. Koonz.

Clinton H. Leighton.

Wm. R. Longenecker.

William D. Lubitz.

John A. Marr.

William B. Martin.

Clarence M. Master.

Carl F. C. Mehlig.

Max. J. G. A. Meibauer.

William H. Meyer, B.A.

Arthur H. Merritt.

Arthur P. Morgan.

John H. McArdle.

George A. McEachren.

Harrison A. McNear.

Kelsey B. Osmun.

George B. Palmer.

David A. Proctor.

William B. Ready.

John W. Remer.

George E. Reynolds.

Joaquin Yela, Jr.

Austin J. Ruddy, Jr.

Mozart M. Solomon, B.A.

Harry L. Sanford.

Johann F. Schaeffer.

Granville E. Scofield.

Otto Schreiber.

Charles A. Secord.

John F. Shea.

Robert A. Sheppard, Jr.

William B. Short, Jr.

Morris Skalmer.

Edgar E. Sloss.

Walter D. Smith.

Oliver E. Stedman.

Howard M. Stowitts.

George T. Stevens.

Albert J. Todd.

Robert V. Totten.

Jason H. Tuttle, Jr.

Clarence W. Travis.

Herbert M. Vanderbilt.

Theodore D. Van Sickel.

Jose. V. Y. Vilaret.

E. Waller.

Frederick S. Welden.

Dwight O. Whedon.

Augustus H. Xiques.

### Missouri Dental College.

The twenty-ninth annual commencement exercises of the Missouri Dental College (Dental Department of Washington University) were held on Thursday, March 28, 1895.

The valedictory was delivered by Dr. Frank A. Glasgow.

The number of matriculates for the session was eighty-six.

The degree of D.D.S. was conferred by Chancellor Winfield S. Chaplin on the following graduates:

| NAME. | RESIDENCE. | NAME. | RESIDENCE. |
|---|---|---|---|
| Samuel F. Arthur | Missouri | Nova G. Phillips | Missouri |
| Harry F. D'Oench | Missouri | Rudolph L. Schmitt | Texas |
| James A. Henderson | Missouri | Clay V. Sidener | Missouri |
| Isaac F. Hereford | Missouri | William F. A. Schultz | Kansas |
| James B. Houf | Missouri | Benjamin F. Stevens. | Missouri |
| William J. Lark | Illinois | Charles I. Trimble | Missouri |
| Lorenz E. Lehmberg | Missouri | Alfred C. Washington | Illinois |
| James F. McLellan | Missouri | Frank C. Wick | Missouri |
| John W. Marsh | Illinois | Thomas W. Willard | Mexico |
| George L. Miller | Missouri | Edward A. Woelk, Jr | Illinois |
| John B. Norris | Missouri | Francis M. Worley | Kansas |
| Thomas Owings, Jr. | Missouri | James McH. Young | Missouri |

### Indiana Dental College.

The sixteenth annual commencement exercises of the Indiana Dental College were held in the Grand Opera House, Indianapolis, on the evening of March 27, 1895.

The number of matriculates during the past session was one hundred and twenty-six.

The following students were graduated:

| NAME. | RESIDENCE. | NAME. | RESIDENCE. |
|---|---|---|---|
| Carl Bauer | Ohio | Minnie Masters | Indiana |
| H. G. Conklin | Ohio | Otto Michaels | Indiana |
| S. S. Curry | Florida | L. M. Mains | Indiana |
| I. W. Carey | Ohio | W. W. Mitchell | Indiana |
| Jesse Emerson | Indiana | J. F. McDonald | Nova Scotia |
| R. J. Gillespie | Indiana | W. E. Pruner | Nebraska |
| E. H. Grace | Indiana | M. D. Powell | Indiana |
| B. M. Harbison | Indiana | P. C. Reade | Indiana |
| F. A. Hamilton | Indiana | H. E. Strain | Indiana |
| B. F. Johnson | Ohio | W. M. Van Scoyoc | Illinois |
| L. L. Kimerer | California | F. H. Walters | Michigan |
| H. S. Lee | Illinois | C. H. Weeks | Ohio |
| M. E. LeGally | Ohio | J. G. Wherry | Indiana |
| C. C. Murphy | Indiana | | |

Total 27.

### Chicago College of Dental Surgery.

The thirteenth annual commencement exercises of the Chicago College of Dental Surgery were held in the Schiller Theater, Chicago, Ill., on Tuesday, April 2, 1895.

The number of matriculates for the session was four hundred and sixteen.

The degree of D.D.S. was conferred on the following graduates by Truman W. Brophy, M.D., D.D.S., dean of the faculty:

Cassius L. Anderson.

Robert E. Anderson.

Rene Anema.

Arthur Atkinson.

George F. Baker.

Peter T. Barber.

Uri N. Barber.

William O. Barrett.

Preston G. Beirman.

Joseph Bendit.

Howell Boatner.

Ivory L. Bowman.

Louis B. Bradley.

John F. Brenneman.

Edward F. Brown.

James J. Carey.

Harry J. Combs.

William R. Cone.

Charles I. Cox.

Edward C. Crawford.

Dexter H. Davison.

Walter Dean.

Walter L. Dickson.

Robert Y. Dudley.

Wallace E. Eddy.

Adney J. Elmer.

George E. Everett.

Albert M. Farr.

Charles H. Farrand.

William Fraser.

Arthur L. Fouche.

Wilber E. Fribley.

Frank H. Gehbe.

Henry W. Glantz.

Alexander W. Glass.

Peter G. Godfrey.

Hart J. Goslee.

Spurgeon D. Gostelow.

Wilbur F. Green.

Alvin T. Grove.

Edgar C. Grove.

Alfred Guthrie.

James P. Harper.

John P. Harvey.

William S. Heaton.

William C. Hessler.

Le Roy E. Hughes.

Louis E. Jelinek.

Augustus C. Jacobs.

David H. Kennedy.

Walter H. Kepner.

Benjamin L. Kirby.

Ora L. Kibler.

Ferdinand C. Kloetzer.

Arthur D. Kifner.

Charles W. Lacey.

William F. Lawrenz.

William H. Lee.

George I. Little.

John B. Lucas.

Russell Lyon.

Lewis M. Magill.

John E. Magunson.

Thomas W. McAfee.

Silas E. McDonald.

Chas. D. McDougal.

Ross N. Martin.

Theodore R. Michaelis.

Francis H. Miller.

Arthur W. Morrow.

Marion J. Newman.

Thomas H. O'Neill.

Chas. A. Pankey.

Chas. S. Parker.

Marshall A. Payne.

Walton A. Perkins.

Wilfred E. Perren.

George W. Pfleeger.

Dean R. Phillips.

William A. Pitt.

Samuel H. Pollock.

Edward Reyer.

Louis R. Richardson

Frank H. Robinson.

Walter A. Rothschild.

William R. Russell.

Martin C. Rifan.

Hugh H. Salmon.

Athanasious J. Sanichas.

John T. Search.

Callanus L. Smith.

Archibald C. Snavely.

Geo. W. Snyder.

Albert F. Solliday.

Chas. J. Sowle.

Ira D. Steele.

Geo. A. Sweetnam.

Geo. E. Tanna.

Mathias A. Thomets.

Edmund G. Tompkins.

Charles C. Trailor.

John E. Trombley.

Peter C. S. Weart.

Ralph H. Washburn.

George N. Wilcox.

Joseph A. Wright.

Edward T. L. York.

Total 107.

### Cincinnati College of Dental Surgery.

The annual commencement exercises of the Cincinnati College of Dental Surgery were held at the Y. M. C. A. building, Sinton Hall, Cincinnati, Ohio, on Thursday evening, April 4, 1895.

An address was made by Professor G. S. Junkerman, M.D., D.D.S., and the valedictory was delivered by Mr. William Howard Neff.

The number of matriculates for the session was twenty-six.

The degree of D.D.S. was conferred on the following graduates by Francis B. James, LL.B., president of the board of trustees: Andrew J. Bradford, William H. Gensley, William Lockman, Jr., William C. McCormick, and Walter L. Stevenson, all of Ohio.

